# Gastrointestinal stromal tumors with pseudocystic change mimicking a pancreatic tumor: two case reports

**DOI:** 10.1186/1752-1947-3-7592

**Published:** 2009-05-07

**Authors:** Ursula Pauser, Sebastian Hinz, Hartmut Merz, Alfred C Feller

**Affiliations:** 1 Department of Pathology, University of Lübeck, Lübeck, Germany; 2 Department of Surgery, University of Kiel, Kiel, Germany

## Abstract

**Introduction:**

Cystic lesions of the upper abdomen normally develop from pancreatic tissue. The differential diagnoses include neoplastic and non-neoplastic lesions. Pseudocystic tumors that secondarily involve the pancreas are very rare and may lead to diagnostic pitfalls.

**Case presentation:**

A 51-year-old woman and a 65-year-old man, both German, presented with abdominal cystic lesions suspected to be pancreatic pseudocysts. Both tumors were classified as gastrointestinal stromal tumors, epithelioid subtype. In one case, tumor origin in the gastric wall was confirmed by relaparotomy. In the other case, a point mutation in *PDGFRalpha* gene, exon 18 proved the diagnosis of gastrointestinal stromal tumor. The tumors were resected and both patients are still alive and disease-free.

**Conclusions:**

The differential diagnosis of cystic lesions in the upper abdomen must include gastrointestinal stromal tumors with pseudocystic change. The origin of a large cystic gastrointestinal stromal tumor may be difficult to determine. Epithelioid tumor pattern, weak or absent KIT expression and detection of *PDGFRalpha* mutation are typical diagnostic parameters of gastric gastrointestinal stromal tumors.

## Introduction

Cystic lesions of the pancreas are usually pseudocysts, related to chronic pancreatitis. Some are inflammatory pseudotumors with regressive cystic change; others are solid-pseudopapillary tumors or serous or mucinous cystic neoplasms of the pancreas.

The diagnostic spectrum of pancreatic cystic lesions has been well investigated. Recently, we introduced a new entity entitled solid and cystic pancreatic hamartomas with a review of the literature [1]. The diagnoses and malignant potential of cystic lesions are sometimes difficult to predict, but in general, they are simply classified by their histology. The following cases demonstrate pancreatic cystic lesions that secondarily involved the pancreas. The histological, immunohistochemical and molecular investigation led to the diagnosis of gastrointestinal stromal tumor (GIST) with pseudocystic change. The difficulties of tumor diagnosis and tumor origin are discussed.

## Case presentation

### Case 1

**Figure 1:**
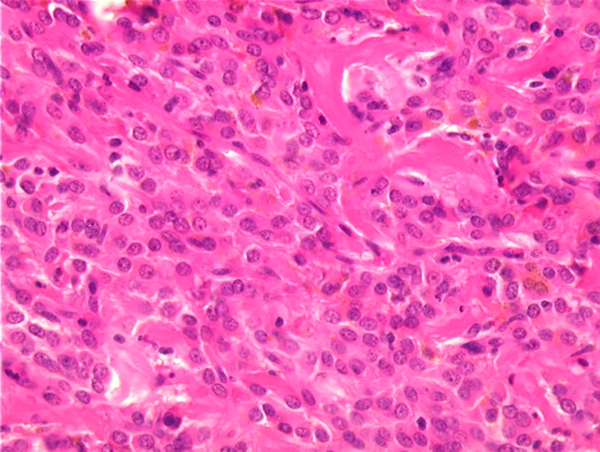
**Epithelioid gastrointestinal stromal tumor with sclerosis, an interspersed chronic lymphocytic inflammatory infiltrate and hemosiderin**. Tumor cells with round to oval nuclei, discrete nucleoli and a moderately broad rim of eosinophilic cytoplasm (hematoxylin and eosin staining; case 1).

A 51-year-old woman presented with a palpable abdominal mass on physical examination. From a computed tomography (CT) scan, a cystic tumor, 20 cm in diameter, was notes to be present between the stomach and the pancreas. With endosonography, the pancreas was inhomogeneous like chronic pancreatitis. Suspected of being a pancreatic pseudocyst, the mass was resected. Necrotic material and multiple solid nodules up to 3 cm in diameter were present. Histological examination revealed an epithelioid neoplasm, resembling a neuroendocrine tumor. Immunohistochemical analysis indicated the characteristic antigen expression pattern of mesenchymal tumors (positivity of vimentin). A postoperative control CT detected a residual tumor. Relaparotomy was performed with complete tumor resection and wedge resection of the gastric wall. The resected specimen demonstrated multiple 0.5 cm gray tumor nodules within a 4 cm in diameter hemorrhagic mouldered area. Histologically, the solid tumor component was well circumscribed and multilobated with fibrous septae, extensive sclerosis, diffuse hemorrhage and pseudocystic degeneration. The histological growth pattern was predominantly epithelioid (Figure 1). The tumor cells showed centrally located round to oval nuclei with discrete nucleoli and a moderately broad rim of eosinophil cytoplasm. Mitoses were very rare (1/50 HPF). Some apoptotic figures were seen. The tumor was well vascularized with an interspersed chronic lymphocytic inflammatory infiltrate adjacent to regular pancreatic tissue. The tumor cells showed strong cytoplasmic staining for the immunohistochemical markers vimentin and CD34 and focally and weak reaction for KIT. Cytokeratin and the neuroendocrine marker synaptophysin were not detected. Expression of the neural marker S100 protein and smooth muscle marker SMA was also lacking. Proliferative activity, identified by Ki-67, was 5%. Sequence analyses of *c-kit* exons 9 and 11 and *PDGFRalpha* exons 12 and 18 were carried out. A mutation was not detected.

### Case 2

**Figure 2:**
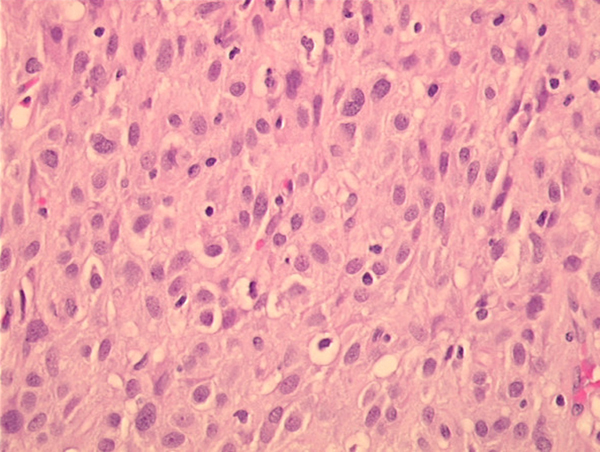
**Epithelioid gastrointestinal stromal tumor with a mild lymphocytic infiltrate (hematoxylin and eosin staining; case 2)**.

**Figure 3:**
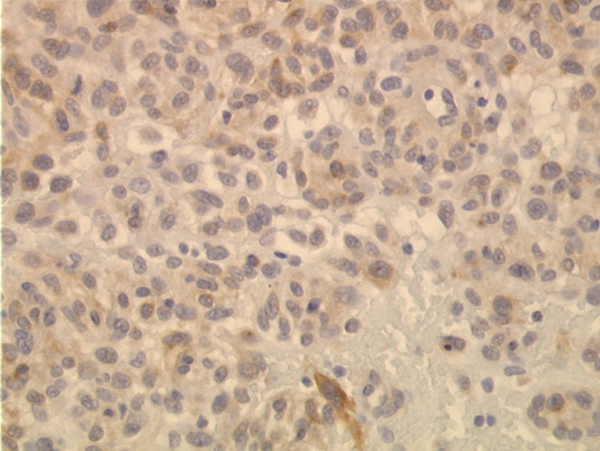
**Weak cytoplasmic expression of KIT with single paranuclear dots (case 2)**. A-C: original magnification ×400.

A 65-year-old man with slight abdominal discomfort was examined by ultrasonography, which revealed a well-demarcated pancreatic cystic tumor, 10 cm in diameter. Three nodules were resected for histological examination. The specimens were solid, gray-brown and up to 3 cm in diameter. The tumor presented an epithelioid growth pattern (Figure 2) with necrosis, hemorrhage and a chronic inflammatory infiltrate. The mitotic rate was 1/50 HPF. Immunohistochemical co-expression of vimentin, CD34 and BCL-2 was detected. The reaction product of KIT was weak, but typically cytoplasmatic dots were seen (Figure 3). The proliferative activity detected with Ki-67 was 5%. Other epithelial and mesenchymal markers were negative (for details, see Table 1). Sequence analyses of *c-kit* and *PDGFRalpha* gene revealed a point mutation in *PDGFRalpha* exon 18 (D842 V). These results confirmed the diagnosis of GIST.

**Table 1:** Immunohistochemical panel with staining results in case 1 and case 2

Antigen	Working dilution	Mono-, polyclonal	Source	Case 1	Case 2
Vimentin	1.50	M	Dako	Pos	Pos
KIT	1:50	P	Dako	Pos	Pos
CD34	1:500		Immunotech, Marseille, France	Pos	Pos
Bcl-2	1:25	P	Dako	Pos	Pos
Synaptophysin	1:20	M	Dako	Neg	Neg
CAM5.2	1:10	M	Becton Dickinson, Franklin Lakes, NJ, USA	Neg	Neg
SMA	1:20		Dako	Neg	Neg
S100	1:500	P	Dako	Neg	Neg
Ki-67	1:100		Dako	5%	5%

Five months later, control CT found a 5.3 cm diameter well demarcated inhomogeneous cystic mass projecting into the lesser sac. Relaparotomy located the residual tumor between the stomach and pancreas. Infiltration of the digestive tract was not observed. The tumor was resected completely. There was a multipart mouldered gray-brown mass 3 cm in diameter. Histological diagnosis confirmed residual epithelioid GIST with pseudocystic change.

## Discussion

The two cases presented here were clinically diagnosed as pancreatic cystic lesions. They were suspected of being pancreatic pseudocysts, due to chronic pancreatitis. Histologically, they showed a solid epithelioid growth pattern resembling a pancreatic neuroendocrine tumor, but lacked epithelial and neuroendocrine markers in the immunohistochemistry. A pancreatic carcinoma was excluded. Instead, the tumors revealed mesenchymal differentiation, but well known mesenchymal entities of the pancreas, that is, leiomyosarcoma or malignant peripheral nerve sheath tumor, were not shown on immunohistochemistry [2]. Moreover, there was no evidence for an inflammatory pseudotumor as discussed earlier [3]. Co-expression of CD34, KIT and BCL-2 characterizes the diagnosis of GISTs and extragastrointestinal stromal tumors (EGISTs). The latter are a small subset (5%) of GISTs that originate outside the gastrointestinal tract. EGISTs are predominantly located in the omentum and mesentery (80%), but also in the retroperitoneum [4,5]. However, a GIST of the pancreas with infiltration of the duodenal wall has been reported [6]. We argue that this tumor, which was 10 cm in diameter, might resemble a gastric or duodenal GIST, which infiltrated the pancreas secondarily. The origin was not proven definitely. Doctors should be wary of diagnosing a primary GIST of the pancreas, especially when the tumor is large and a gastric or intestinal GIST is not excluded.

In our two cases, epithelioid growth pattern, weak KIT expression and low proliferative activity was suspicious for gastric GIST. In case one, the tumor origin in the stomach was confirmed at relaparotomy. In case two, a point mutation in *PDGFRalpha* exon 18 strengthened the diagnosis of GIST, evolving in the stomach, since this mutation was detected predominantly in gastric GISTs with epithelioid growth pattern [7]. Moreover, this peculiar tumor subtype has a low mitotic rate and favorable prognosis [7,8], as seen in our two cases. Both GISTs were resected completely and the patients have had a relapse-free follow-up so far (8 and 3 years). Based on the low mitotic rate (1 Mitosis/50 HPF), we classified the tumor risk of malignancy as low to intermediate according to Fletcher's criteria [9]. The prognostic marker tumor size must be discussed. The large tumors measured with ultrasound (20 cm and 10 cm in diameter, respectively) included a cystic appearance. In contrast to this, the solid tumor part of the resected specimen was considerably less than 10 cm in diameter (about 4 cm in case 1 and 5 cm in case 2). The clinically described cysts were due to regressive changes of tumor tissue and are termed pseudocysts. The tumor was typically necrotic and hemorrhagic. An epithelial lining as defined in cysts was not seen.

## Conclusions

The differential diagnoses of pancreatic cystic lesions must include GISTs with pseudocystic changes. The origin of large GISTs in the upper abdomen may be difficult to determine, especially when located between the stomach, duodenum and pancreas. A weak or absent expression of KIT should not exclude the diagnosis of GIST, since this is characteristic for gastric GISTs with epithelioid differentiation. Mutation analyses are helpful in such cases.

## Consent

Written informed consent was obtained from the patients for publication of these case reports and any accompanying images. A copy of the written consent is available for review by the Editor-in-Chief of this journal. The retrospective study was undertaken in accordance with institutional guidelines. Informed consent for scientific evaluation had been obtained from patients at their primary clinical treatment.

## Competing interests

The authors declare that they have no competing interests.

## Authors' contributions

UP drafted the manuscript, carried out the immunohistochemical staining, the molecular analysis, researched the literature and took the photographs. SH contributed the clinical data. HM and AF revised the manuscript critically for important intellectual content. All authors read and approved the final manuscript.
